# Aktuelle Diagnostik und Behandlung der Sprunggelenks-Distorsion in Deutschland

**DOI:** 10.1007/s00113-024-01428-y

**Published:** 2024-04-18

**Authors:** Philipp Lichte, Christian Weber, Michael Otto, Felix Bläsius, Natalia Gutteck, Frank Hildebrand, Dariusch Arbab

**Affiliations:** 1 Klinik für Orthopädie, Unfall- und Wiederherstellungschirurgie, Uniklinik RWTH Aachen, Pauwelsstr. 30, 52074 Aachen, Deutschland; 2 Klinik für Orthopädie, Universitätsklinikum Halle (Saale), Halle (Saale), Deutschland; 3 Klinik für Orthopädie/Unfallchirurgie, St. Elisabeth-Hospital Herten, Mitglied der Medizinischen Fakultät der Universität Witten/Herdecke, Herten, Deutschland

**Keywords:** Sprunggelenksverletzung, Bandruptur, Ottawa Ankle Rules, Syndesmose, Außenband, Upper ankle injury, Ligament tear, Ottawa ankle rules, Syndesmosis, Lateral ligament

## Abstract

**Hintergrund:**

Die Distorsion des oberen Sprunggelenks (OSG) ist eine der häufigsten Verletzungen des Bewegungsapparates. Das Verletzungsmuster bestimmt die zu wählende Therapie und ist entscheidend für das Outcome. *Die isolierte Ruptur des Außenbandapparates ist die häufigste strukturelle Verletzung.* Hierfür wird leitliniengerecht eine konservative Therapie empfohlen, für Kombinationsverletzungen besteht hingegen hinsichtlich des diagnostischen und therapeutischen Vorgehens keine einheitliche Vorgehensweise. Ziel der bundesweiten Umfrage war es, einen Überblick über die aktuelle diagnostische Strategie und die gängigen therapeutischen Konzepte in Deutschland zu gewinnen.

**Material und Methoden:**

Mitglieder der Deutschen Gesellschaft für Orthopädie und Unfallchirurgie (DGOU) wurden eingeladen, an einer online Umfrage zum diagnostischen und zum therapeutischen Vorgehen bei OSG-Distorsion mit einem Fragebogen, bestehend aus 20 Fragen, teilzunehmen. Neben Fragen zur Fachrichtung und zum Tätigkeitsfeld wurden die Teilnehmer gebeten, ihr diagnostisches und therapeutisches Vorgehen darzustellen.

**Ergebnisse:**

Insgesamt nahmen 806 Teilnehmer an der Umfrage teil. Die Mehrzahl der Befragten waren Orthopäden und Unfallchirurgen und in der Klinik tätig. Bei der Erstvorstellung werden der Schubladen Test (89,5 %) und der Inversions‑/Eversionstest (81,6 %) am häufigsten durchgeführt. Eine Röntgenuntersuchung führen 88,1 % regelhaft bei Erstvorstellung durch, 26,5 % auch eine sonographische Untersuchung. Die isolierte Verletzung des Lig. fibulotalare anterius (LFTA) behandeln 99,7 % konservativ, in 78,8 % der Fälle mit Vollbelastung in der Orthese. Die vollständige Außenbandruptur würden 79,9 % konservativ behandeln. Eine kombinierte Außenbandruptur mit Syndesmosenverletzung würden 30,1 % der Befragten konservativ behandeln.

**Diskussion:**

Aufgrund der Heterogenität der Verletzungsmuster nach einer OSG-Distorsion bestehen keine einheitlichen diagnostischen und therapeutischen Behandlungsempfehlungen. Die Ottawa Ankle Rules und die Sonographie werden trotz der guten Studienlage nur wenig eingesetzt. Die isolierte LFTA-Ruptur wird von einem Großteil der Befragten leitliniengerecht diagnostiziert und behandelt. Bei kombinierten Verletzungen des Innen- und Außenbandapparates entscheidet sich die Mehrheit für ein konservatives Vorgehen, was durch die Literatur bei geringer Evidenz als gerechtfertigt erscheint. Kombinierte Verletzungen der Syndesmose und des Außenbandapparates würde die Mehrzahl der Befragten operativ behandeln, was ebenfalls mit den Empfehlungen der Literatur korreliert. Der Versorgungsstandard in Deutschland entspricht somit weitgehend den Literaturempfehlungen.

**Graphic abstract:**

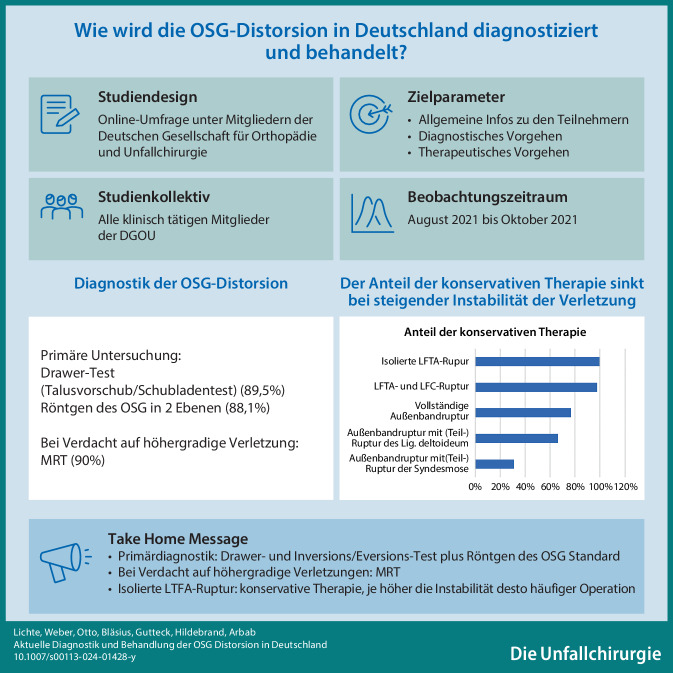

## Abstract (en)

**Background:**

Ankle sprains are one of the most frequent injuries of the musculoskeletal system. The injury pattern determines the treatment and are crucial for the outcome. Nonoperative treatment is commonly recommended for isolated injuries of the lateral ligaments but no standard strategy exists in combined ankle ligament injuries. The goal of this national survey was to achieve an overview about the current diagnostic strategies and common treatment concepts in Germany.

**Material and methods:**

All members of the German Society for Orthopaedics and Trauma Surgery (DGOU) were invited to participate in an anonymous survey about the diagnostic and therapeutic approach in cases of ankle sprains. The online survey consisted of 20 questions. Besides questions about the speciality and scope of activities the participants were ask to depict their diagnostic and therapeutic strategy.

**Results:**

A total of 806 participants completed the survey. Most of them were orthopedic trauma surgeons and worked in a hospital. During the first presentation the anterior drawer test (89.5%) and the inversion/eversion test (81.6%) were most commonly used, 88.1% always make an X‑ray examination and 26.5% an ultrasonography examination. Isolated injuries of the anterior fibulotalar ligament (LFTA) were treated nonoperatively by 99.7% of the participants, 78.8% recommend full weight bearing in an orthesis, 78.8% treat the complete rupture of the lateral ligaments without operation whereas 30.1% stated that they would treat a combined lateral ligaments rupture with an injury of the syndesmosis nonoperatively.

**Discussion:**

Due to the heterogeneity of injury patterns after ankle sprain no consistent recommendations for diagnostics and treatment exist. The Ottawa ankle rules and ultrasonography were not often utilized despite of the good evidence. The isolated rupture of the LFTA is diagnosed and treated according to the national guidelines by most of the participants. In cases of combined injuries of the lateral and medial ankle ligaments the majority choose a nonoperative treatment strategy which is justified by the guidelines with a low level of evidence. Combined injuries of the syndesmosis and the lateral ankle ligaments were treated operatively, which also correlates with the recommendations in the literature. The standard care of ankle sprain in Germany is in accordance with the recommendations from the current literature.

## Hintergrund

Der Begriff OSG-Distorsion beschreibt die Verdrehung des oberen Sprunggelenkes (OSG), also einen Verletzungsmechanismus. Dennoch wird die OSG-Distorsion im allgemeinen Sprachgebrauch häufig auch als Diagnose genannt. Sie umfasst dann eine der häufigsten Verletzungen des Bewegungsapparates und wird als häufigste Sportverletzung überhaupt gezählt. Ihr Anteil beträgt bis zu 40 % aller Sportverletzungen. 85 % aller OSG-Distorsionen ereignen sich als Supinationstrauma. Die Verletzung betrifft in den meisten Fällen die lateralen Kapsel-Band-Strukturen, hier v. a. das Lig. fibulotalare anterius (LFTA). In den USA wird für Rupturen des Außenbandapparates von einer Inzidenz von 1/10.000 Personen und Tag ausgegangen.

Zusätzlich zum lateralen Bandapparat können aber weitere Strukturen verletzt sein, z. B. die Syndesmose, der mediale Bandapparat und die Peronäalsehnen. Auch knöcherne Begleitverletzungen (z. B. die Fraktur des Processus lateralis tali) oder Knorpelläsionen können auftreten. *Die Verletzungsschwere wird in der klinischen Praxis nicht selten unterschätzt.*

*Das Erkennen des genauen Verletzungsmusters ist jedoch essenziell, um spätere Komplikationen (z.* *B. chronische Instabilitäten) zu vermeiden.* Hierfür stehen verschiedene klinische Untersuchungen und Möglichkeiten der Bildgebung, deren Einsatz teils uneinheitlich gehandhabt wird, zur Verfügung.

Während für die isolierte Therapie der Verletzungen des Außenbandes eine konservative funktionelle Therapie in den gängigen Leitlinien empfohlen wird, ist bei Kombinationsverletzungen die Therapie individuell sehr unterschiedlich.

Ziel dieser Arbeit war es, einen Überblick über die aktuelle diagnostische Strategie und die gängigen therapeutischen Konzepte in Deutschland zu gewinnen.

## Materialien und Methoden

Die Mitglieder der Deutschen Gesellschaft für Orthopädie und Unfallchirurgie (DGOU) wurden zwischen August 2021 und Oktober 2021 per E‑Mail eingeladen, an einer anonymen Umfrage zum diagnostischen und zum therapeutischen Vorgehen bei OSG-Distorsionen teilzunehmen. Die Umfrage wurde über eine Cloud-basierte Software (https://www.surveymonkey.com/) an alle Mitglieder versendet. Im Abstand von 6 Wochen erfolgte eine Erinnerungs-Mail. Nach weiteren 2 Wochen wurde die Umfrage dann geschlossen. Die Versendung des Fragebogens erfolgte ausschließlich über die Fachgesellschaft, sodass die genauen Empfänger den Autoren nicht bekannt sind. Die beantworteten Fragebogen sind anonymisiert gesammelt worden.

Der eigens für diese Umfrage erstellte Fragebogen umfasste 20 Fragen zu 3 Kategorien (Tab. 1 und 2):

- Allgemeine Informationen mit Angaben zur Fachrichtung (Orthopädie und Unfallchirurgie, Orthopädie, Unfallchirurgie, Chirurgie), Tätigkeitsfeld (Klinik, Praxis) u. a.
- Diagnostisches Vorgehen (klinische Untersuchung, bildgebende Diagnostik u. a.)
- Therapeutisches Vorgehen (konservativ, operativ u. a.)

**Table 1:** 

Frage	Antwortmöglichkeiten	
In welcher Fachrichtung sind Sie tätig?	Orthopädie	
	Unfallchirurgie	
	Orthopädie und Unfallchirurgie	
	Allgemeinchirurgie	
Wie lange sind Sie bereits klinisch tätig?	> 6 Jahre	
	6–10 Jahre	
	11–15 Jahre	
	16–20 Jahre	
	> 20 Jahre	
Wo sind Sie tätig?	Universitätsklinikum	
	Krankenhaus der Maximalversorgung	
	Krankenhaus der Grundversorgung	
	Praxis(-klinik)	
Ist in Ihrer Klinik/Abteilung ein zertifizierter Fußchirurg/eine zertifizierte Fußchirurgin tätig?	Ja, ich bin zertifizierte Fußchirurgin/Fußchirurg	
	Ja, ein Kollege/eine Kollegin	
	Nein	
Wie viele OSG-Distorsionen werden in Ihrer Klinik/Praxis pro Jahr behandelt?	1–25	
	25–50	
	51–100	
	101–200	
	> 200	
Welche der folgenden klinischen Tests führen Sie im Rahmen der Erstvorstellung regelhaft durch? (Mehrfachnennung möglich)	Inversions‑/Eversions-Stress-Test	
	Drawer-Test (Talusvorschub)	
	Frick-Test	
	Squeeze-Test	
	Crossed-Leg-Test	
Wie hilfreich finden Sie die Ottawa Ankle/Foot Rules für die Entscheidungsfindung zur Röntgendiagnostik?	Sehr hilfreich	
	Hilfreich	
	Manchmal hilfreich	
	Wenig hilfreich	
	Nicht hilfreich	
Welche Diagnostik führen Sie regelhaft im Rahmen der Erstvorstellung nach dem Distorsionsereignis durch? (Mehrfachnennung möglich)	Röntgen ohne Belastung	
	Röntgen im Stand	
	Gehaltene Aufnahmen	
	Sonographie	
	MRT	
	CT	
	DVT	
	Andere	
Welche zusätzliche Diagnostik führen Sie bei Verdacht auf höhergradige Verletzungen regelmäßig durch? (Mehrfachnennung möglich)	Röntgen im Stand	
	Gehaltene Aufnahmen	
	Sonographie	
	MRT	
	CT	
	DVT	
	Andere	
Wie schätzen Sie den Nutzen der folgenden Untersuchungsmethoden bei Verdacht auf höhergradige Verletzungen ein?	Röntgen im Stand	Jeweils:
	Gehaltene Aufnahmen	Sehr hilfreich
	MRT	Hilfreich
	CT	Manchmal hilfreich
	DVT	Wenig hilfreich
		Nicht hilfreich

**Table 2:** 

Fragen	Antwortmöglichkeiten
Welche Therapie würden Sie bei einer isolierten Ruptur des Lig. fibulotalare anterius (LFTA) bevorzugen?	Konservativ
	Operative Rekonstruktion des LFTA
	Andere Technik
Wie erfolgt die Nachbehandlung?	Unterschenkelgips/Walker mit Entlastung
	Unterschenkelgips/Walker mit Teilbelastung
	Unterschenkelgips/Walker mit Vollbelastung
	OSG-Orthese mit Teilbelastung
	OSG-Orthese mit Vollbelastung
	Taping
	Andere
Welche Therapie würden Sie bei einer kombinierten Ruptur des LFTA und des Lig. fibulocalcaneare (LFC) im Rahmen eines Erstereignisses einer OSG-Distorsion bevorzugen?	Konservativ
	Operative Rekonstruktion des LTFA
	Operative Rekonstruktion des LFC
Wie erfolgt die Nachbehandlung?	Unterschenkelgips/Walker mit Entlastung
	Unterschenkelgips/Walker mit Teilbelastung
	Unterschenkelgips/Walker mit Vollbelastung
	OSG-Orthese mit Teilbelastung
	OSG-Orthese mit Vollbelastung
	Taping
	Andere
Welche Therapie würden Sie bei einer vollständigen Ruptur des Außenbandapparates im Rahmen eines Erstereignisses einer OSG-Distorsion bevorzugen?	Konservativ
	Operative Rekonstruktion des LTFA
	Operative Rekonstruktion des LFC
	Operative Rekonstruktion des LTFP
	Andere Technik
Wie erfolgt die Nachbehandlung?	Unterschenkelgips/Walker mit Entlastung
	Unterschenkelgips/Walker mit Teilbelastung
	Unterschenkelgips/Walker mit Vollbelastung
	OSG-Orthese mit Teilbelastung
	OSG-Orthese mit Vollbelastung
	Taping
	Andere
Welche Therapie würden Sie bei einer kombinierten Ruptur des Außenbandapparates mit einer (Teil‑)Ruptur des Lig. deltoideum im Rahmen eines Erstereignisses einer OSG-Distorsion bevorzugen?	Konservativ
	Operative Rekonstruktion des LTFA
	Operative Rekonstruktion des LFC
	Operative Rekonstruktion des LTFP
	Operative Rekonstruktion des Lig. deltoideum
	Andere Technik
Wie erfolgt die Nachbehandlung?	Unterschenkelgips/Walker mit Entlastung
	Unterschenkelgips/Walker mit Teilbelastung
	Unterschenkelgips/Walker mit Vollbelastung
	OSG-Orthese mit Teilbelastung
	OSG-Orthese mit Vollbelastung
	Taping
	Andere
Welche Therapie würden Sie bei einer kombinierten Ruptur des Außenbandapparates mit einer (Teil‑)Ruptur der Syndesmose im Rahmen eines Erstereignisses einer OSG-Distorsion bevorzugen?	Konservativ
	Operative Rekonstruktion des LTFA
	Operative Rekonstruktion des LFC
	Operative Rekonstruktion des LTFP
	Operative Rekonstruktion der Syndesmose
	Andere Technik
Wie erfolgt die Nachbehandlung?	Unterschenkelgips/Walker mit Entlastung
	Unterschenkelgips/Walker mit Teilbelastung
	Unterschenkelgips/Walker mit Vollbelastung
	OSG-Orthese mit Teilbelastung
	OSG-Orthese mit Vollbelastung
	Taping
	Andere

In der vorliegenden Arbeit werden ausgewählte Items präsentiert. Die Fragebogen wurden in einer univariaten Datenanalyse mit der Angabe von numerischen Häufigkeiten und der prozentualen Verteilung ausgewertet und entsprechend grafisch dargestellt. Die Auswertung erfolgte anhand aller beantworteten Fragen einer Kategorie.

Ein Ethikantrag war nicht erforderlich, da keine Patientendaten erhoben wurden.

## Ergebnisse

Es nahmen insgesamt 806 Teilnehmer an der Befragung teil. Der erste Teil der Umfrage zur Diagnostik wurde von 740 Teilnehmern vollständig ausgefüllt, der zweite Teil zur Therapie von 685 Teilnehmern.

**Figure 1:**
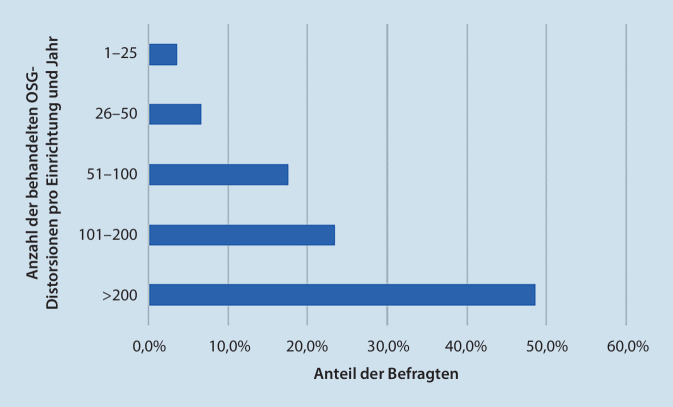

Die Mehrzahl der Befragten ist in einer Einrichtung mit kombinierter Orthopädie und Unfallchirurgie tätig (463; 57,4 %). Die meisten Teilnehmer sind bereits langjährig klinisch tätig (59,6 % seit mindestens 16 Jahren). Während sich knapp zwei Drittel auf die Krankenhäuser der unterschiedlichen Versorgungsstufen verteilen, geben 34,6 % eine Praxis als Tätigkeitsumfeld an. 18,9 % der Teilnehmer sind selbst zertifizierte Fußchirurgen, und weitere 34 % arbeiten mit einem zertifizierten Fußchirurgen zusammen. Im Median werden in den Einrichtungen der Befragten 101 bis 200 OSG-Distorsionen im Jahr behandelt, bei 48,6 % mehr als 200 Fälle/Jahr (Abb. 1).

In der klinischen Untersuchung im Rahmen der Erstvorstellung werden der Drawer-Test (Talusvorschub/Schubladentest) (89,5 %) und der Inversions‑/Eversions-Stress-Test (81,6 %) am häufigsten durchgeführt. Für die Entscheidungsfindung zur Röntgendiagnostik fanden 43,3 % die Ottawa Ankle Rules hilfreich, 27,3 % manchmal hilfreich und 29,4 % wenig oder nicht hilfreich. 88,1 % gaben an, *dass sie im Rahmen der Erstvorstellung regelhaft ein Röntgenbild ohne Belastung anfertigen würden, ohne dass dies im Rahmen des Fragebogens weiter präzisiert wurde.* Zweithäufigste Diagnostik ist die Sonographie (26,5 %), gefolgt von Röntgenbildern im Stand (10,4 %). Bei *klinischem *Verdacht auf höhergradige Verletzungen führen 90 % der Befragten eine Magnetresonanztomographie(MRT)-Untersuchung durch, 37,2 % eine Computertomographie (CT). Dies spiegelt sich auch darin wider, dass 91,6 % die MRT für sehr hilfreich oder hilfreich bei Verdacht auf höhergradige Verletzungen einschätzen. Die CT wird von 51,8 % für mindestens hilfreich gehalten. Die digitale Volumentomographie (DVT) wird nur von 16,9 % als hilfreich oder sehr hilfreich eingeschätzt, gehaltene Aufnahmen von 14,3 %.

Bei Vorliegen einer isolierten LFTA-Ruptur präferieren 99,7 % aller Befragten die konservative Therapie, wobei 78,7 % eine OSG-Orthese mit Vollbelastung anlegen würden, 22,2 % eine OSG-Orthese mit Teilbelastung und 10,8 % ein Taping durchführen würden.

Bei einer kombinierten Ruptur des LFTA und des Lig. fibulocalcaneare (LFC) würden 98 % eine konservative Therapie bevorzugen, wobei 50,4 % eine OSG-Orthese mit Vollbelastung anlegen würden, 37,5 % eine OSG-Orthese mit Teilbelastung. 13,9 % würden eine Ruhigstellung im Unterschenkelgips oder Walker mit Teilbelastung empfehlen.

Bei einer vollständigen Ruptur des Außenbandapparates würden 79,9 % eine konservative Therapie bevorzugen, 22,9 % würden das LFTA rekonstruieren und 21 % das LFC. Die Nachbehandlung würde bei 33 % in einem Unterschenkelgips oder Walker mit Teilbelastung, bei 28,5 % in einer OSG-Orthese mit Vollbelastung und bei 28,3 % mit einer OSG-Orthese und Teilbelastung erfolgen.

Liegt eine Kombination aus einer vollständigen Außenbandruptur und einer Verletzung des Innenbandes vor, so würden dies 66,7 % konservativ behandeln. 31 % würden das Innenband operativ rekonstruieren und 22,9 % alternativ oder zusätzlich das LFTA. Die Nachbehandlung würden 41,8 % im Unterschenkelgips/Walker mit Teilbelastung gestalten, 22,7 % würden eine OSG-Orthese mit Teilbelastung bevorzugen und 22,5 % eine Ruhigstellung mit Entlastung.

**Figure 2:**
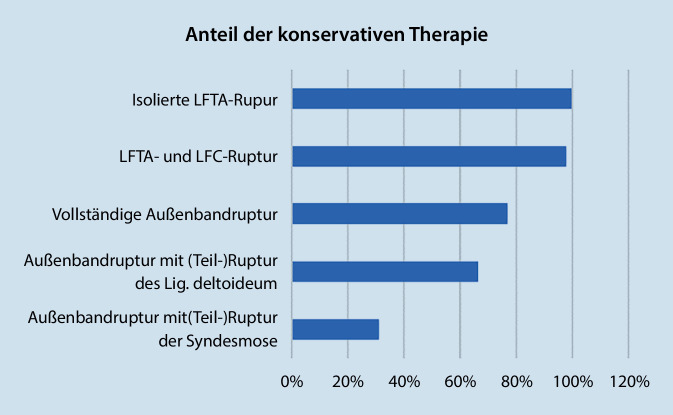

Eine Kombination aus Außenbandruptur mit einer Syndesmosenverletzung würden nur 30,1 % konservativ behandeln. 69,9 % würden die Syndesmose rekonstruieren, mehrheitlich durch Implantation von einer bis 2 Stellschrauben. 17,5 % würden zusätzlich eine Rekonstruktion des LFTA durchführen. Die Nachbehandlung ist bei 90,4 % der Befragten eine Ruhigstellung im Unterschenkelgips/Walker. 41,3 % empfehlen die vollständige Entlastung, 44,4 % eine Teilbelastung (Abb. 2).

## Diskussion

Patienten mit einer OSG-Distorsion machen in der Notaufnahme bis zu 20 % des unfallchirurgischen Patientengutes aus [1]. Die Diagnostik und Behandlung von OSG-Distorsionen werden daher in hoher Frequenz im klinischen Alltag durchgeführt. Dies erklärt möglicherweise die überdurchschnittlich hohe Zahl an Teilnehmern bei dieser Umfrage (806 Teilnehmer, *Beantwortungsquote von 9,7* *%, die Quote bei vergleichbaren Umfragen der DGOU liegt bei 5* *%)* [2].

Aus sozioökonomischer Sicht handelt es sich aufgrund der hohen Prävalenz und der damit verbundenen beruflichen Ausfallzeiten um eine bedeutsame Verletzung. Waterman et al. zeigten zumindest eine temporäre Arbeitsunfähigkeit bei bis zu 60 % der Betroffenen [3]. Bis zu 74 % aller Patienten nach OSG-Distorsion berichten zudem über bleibende Einschränkungen, wie z. B. Schmerzen und Schwellung [4]. Insbesondere aufgrund des frühen Altersgipfels für Erstverletzungen von 15 bis 19 Jahren [3, 5] sind daher eine exakte Diagnose und Therapie zur Vermeidung weiterer Distorsionsereignisse, einer chronischen Instabilität und persistierenden Beschwerden besonders wichtig.

In den letzten Jahren haben sich die Empfehlungen zur Therapie der Außenbandrupturen am OSG deutlich vereinheitlicht. In diesem Zusammenhang ist für Deutschland auch die S1-Leitlinie „Frische Außenbandruptur am Oberen Sprunggelenk“ der DGU von 2017 [6] zu sehen, die auf der Basis von Studienergebnissen Empfehlungen zu Diagnostik und Therapie als Expertenkonsens zusammenfasst. Für Kombinationsverletzungen der Bänder des OSG ist die Studienlage als unzureichend einzuschätzen. Daher existieren hierzu auch keine klaren Konsensempfehlungen.

Die vorliegende Studie bietet die erste Übersicht über die aktuelle Versorgungsrealität in der klinischen Diagnostik und Therapie der OSG-Distorsion in Deutschland.

Im Rahmen der klinischen Erstuntersuchung sind v. a. der Drawer-Test und der Inversions‑/Eversions-Stress-Test verbreitet. Dies entspricht auch den Empfehlungen der aktuellen S1-Leitlinie zur frischen Außenbandruptur am oberen Sprunggelenk. Beide weisen in der Literatur eine hohe Sensitivität auf. Für den Drawer-Test wurde eine Sensitivität von 86 % bei einer Spezifität von 74 % beschrieben [7].

Auch eine aktuelle cross-sektionale Studie bewertet den Drawer-Test als nützlich zur Erkennung von Instabilitäten des Sprunggelenkes [8]. Eine Untersuchung 4 bis 7 Tage nach dem Unfall kann beim erfahrenen Untersucher eine noch höhere Sensitivität (96 %) und Spezifität (84 %) aufweisen [9], kann allerdings potenziell auch einen schädigenden Einfluss auf die bereits begonnenen Heilungsvorgänge haben.

Ungefähr jeweils die Hälfte der Befragten nutzt den Frick-Test oder den Squeeze-Test zur Untersuchung der Syndesmose. Eine Testung der Syndesmosenstabilität mittels Squeeze-Test hat in der Literatur eine relativ geringe Sensitivität von 30 % bei einer hohen Spezifität von 93,5 % [10]. Aufgrund der hohen Spezifität wird daher eine Kombination mit anderen Tests empfohlen [11].

Auch die Nutzung der apparativen Diagnostik in unserer Umfrage folgt im Wesentlichen den Empfehlungen der aktuellen Leitlinie. Hier wird das konventionelle Röntgenbild in 2 Ebenen als notwendige Untersuchung empfohlen. Diese wird auch von 88,1 % der Teilnehmer durchgeführt. Bei der Indikationsstellung zur Röntgendiagnostik können die „Ottawa Ankle Rules“ zur Hilfe genommen werden. Diese haben in einer Metaanalyse eine Sensitivität von 99 % zum Ausschluss einer Fraktur gezeigt [12]. Allerdings wurde in einer Validierung an einem deutschen Patientenkollektiv nur eine Sensitivität von 94 % bei einer Spezifität von 17 % gezeigt. Es wurde geschätzt, dass durch eine konsequente Anwendung lediglich 15 % der Röntgenaufnahmen eingespart werden könnten [13]. In der Leitlinie der DGU wird erwähnt, dass ein routinemäßiger Einsatz der „Ottawa Ankle Rules“ in Deutschland nicht üblich ist. Dies spiegelt sich auch in unseren Ergebnissen wider. Nur 43,3 % halten diese für hilfreich oder sehr hilfreich.

Mit 26,5 % ist die Sonographie laut unserer Umfrage ein weiteres eingesetztes Diagnostikum. Van Dijk et al. konnten nachweisen, dass die Sensitivität mindestens vergleichbar mit gehaltenen Röntgenaufnahmen war [9]. Für LFTA-Verletzungen wurde eine Sensitivität von 91 % im Vergleich zur Arthroskopie festgestellt [14]. Kritisch angemerkt wird jedoch die hohe Untersucherabhängigkeit.

Gehaltene Aufnahmen haben in der Diagnostik der akuten OSG-Distorsion keine Bedeutung mehr (2,4 % der Teilnehmer). Auch dies ist kongruent zur Leitlinie.

Bei Verdacht auf eine *höhergradige, über das LFTA hinausgehende* Verletzung hat sich im Rahmen unserer Umfrage die MRT-Diagnostik eindeutig als bevorzugte Methode herausgestellt. Sie wird von 90 % der Befragten durchgeführt. Die MRT-Untersuchung bietet eine dem Röntgen und der CT signifikant überlegene Sensitivität für die Diagnose von Syndesmosenverletzungen [15]. In einer Metaanalyse von 2019 zeigte sich eine gepoolte Sensitivität von 0,929. Im Vergleich dazu zeigte sich für die CT eine Sensitivität von 0,669 [15]. Auch (osteo-)chondrale Läsionen lassen sich mittels MRT mit hoher Sicherheit aufdecken [16], wobei allerdings das korrekte Staging schwieriger erscheint. Lee et al. beschrieben hier nur eine Übereinstimmung von 81 % für das MRT-Ergebnis im Vergleich zur Arthroskopie [17]. Hier könnte eine DVT mit intraartikulärer Kontrastmittelapplikation Vorteile bieten [18]. Allerdings ist die DVT aktuell noch nicht in breitem Einsatz: Nur 2,4 % der Befragten setzen diese bei OSG-Distorsionen ein.

Die laterale Bandruptur ist eine Domäne der konservativen Therapie. 2007 konnten Kerkhoffs et al. in einer Cochrane-Analyse zeigen, dass bei wenig aussagekräftiger Studienlage die operative der konservativen Therapie nicht signifikant überlegen war [19]. Auch in einer prospektiven randomisierten Studie an 51 jungen Männern konnte kein Vorteil für die operative Therapie gezeigt werden [20]. Zu gleich konnte in mehreren Studien gezeigt werden, dass die frühfunktionelle Therapie in einer supinationshemmenden OSG-Orthese *für mindestens 5 Wochen *der immobilisierenden Therapie überlegen ist. Dies bestätigte auch ein systematisches Review von 2017 [21]. Die funktionelle konservative Therapie wird auch von nahezu allen Befragten, zumindest für die Ruptur des LFTA und die kombinierte Ruptur von LFTA und LFC, so gehandhabt. Bei vollständigen Rupturen des lateralen Bandapparates bevorzugen allerdings ungefähr 20 % der Befragten die operative Rekonstruktion des LFTA oder des LFTA und des LFC. Für diese Fälle mit hochgradiger Instabilität empfiehlt die Leitlinie eine individuelle Entscheidung. Allerdings liegt auch hier keine Evidenz zugunsten der operativen Therapie vor. Für Leistungssportler werden besonders gute Ergebnisse mit der nichtoperativen funktionellen Therapie beschrieben, da die muskuläre aktive Stabilisierung des OSG bei diesen besonders effektiv trainiert werden kann [22].

*Kombinierte Rupturen des lateralen und des medialen Bandapparates ohne weitere Begleitverletzungen sind sehr seltene Verletzungen* [23]*. Daher liegt für die Therapie keine ausreichende Evidenz vor. Es wird jedoch in den vorliegenden Fallberichten über tibiotalare Luxationen ohne Frakturen eine geschlossene Reposition und Ruhigstellung beschrieben* [24–26]*.* Tatsächlich wählten auch 66,1 % der Teilnehmer unserer Umfrage die konservative Therapie aus, in der Mehrzahl mit Ruhigstellung im Walker und Ent- oder Teilbelastung. Von den Befragten, die eine operative Therapie wählen würden, würden die meisten das Innenband und/oder das LFTA rekonstruieren. *Dieses Vorgehen wird von den Ergebnissen einer aktuellen Kadaverstudie unterstützt. Brady et al. konnten nachweisen, dass eine Rekonstruktion des vorderen Anteils des Lig. deltoideum einen hohen Einfluss auf die Stabilität des OSG hat *[27]*.*

Relativ deutlich ist das Antwortbild wiederum bei der Therapie einer Kombination aus Außenband- und Syndesmosenverletzung. Hier würden 70 % eine Rekonstruktion der Syndesmose durchführen. Tatsächlich wird dies auch für isolierte Syndesmosenverletzungen mit einer nachgewiesenen Instabilität der Sprunggelenkgabel oder dem Nachweis der Verletzung von mindestens 2 der 3 Bestandteile der Syndesmose empfohlen [28, 29]. Bei isolierten Läsionen des anterior-inferioren tibiofibularen Ligaments (AITFL) (Grad-I- und Grad-II-Läsionen) und geschlossener Sprunggelenkgabel kann eine konservative Therapie mit guten Erfolgsaussichten erfolgen [29, 30]. Bei Grad-II-Läsionen wird von vielen Autoren eine längere Ruhigstellung mit Entlastung empfohlen [31]. Entsprechend diesen Empfehlungen werden von über 80 % der Befragten unserer Umfrage auch eine Ruhigstellung und Ent- oder Teilbelastung als präferierte Nachbehandlung angegeben.

## Limitierung der Studie

Es handelte sich bei der Untersuchung um eine webbasierte anonymisierte Umfrage. Eine mehrfache Teilnahme eines Teilnehmers lässt sich durch diese Methode nicht sicher vermeiden. Zudem wiesen die Teilnehmer eine langjährige klinische Tätigkeit im orthopädisch/unfallchirurgischen Fachgebiet auf. In die Versorgung im Notdienst sind aber auch Ärztinnen und Ärzte aus anderen Fachgebieten und mit weniger Berufserfahrung involviert, deren Einfluss in dieser Studie möglicherweise nicht ausreichend abgebildet wird.

Eine weitere wichtige Limitierung besteht in der Befragung mittels Fragebogen. Hierbei können aufgrund der geschlossenen Fragestellung und Einschränkungen der Antwortmöglichkeiten Ungenauigkeiten entstehen und die Antworten der Befragten beeinflusst werden.

## Schlussfolgerung

Die Distorsion des oberen Sprunggelenkes ist eine sehr häufige Verletzung. Die Herausforderung in der Diagnostik besteht darin, die komplexen Fälle herauszufiltern und einer adäquaten Therapie zuzuführen. Die Ergebnisse unserer Befragung zeigen, dass die eingesetzten diagnostischen Mittel im Wesentlichen den Literaturempfehlungen folgen. Perspektivisch wird sich zeigen, ob die DVT bei größerer Verbreitung und besserer Datenlage einen höheren Stellenwert bekommen wird. Bei der Behandlung von isolierten Außenbandverletzungen konnten wir einen breiten Konsens zur konservativen funktionellen Therapie zeigen. Bei Beteiligung der Syndesmose wurde mehrheitlich eine operative Rekonstruktion der Syndesmose ausgewählt. Insgesamt entspricht damit auch der aktuelle therapeutische Standard den Empfehlungen der S1-Leitlinie der DGU bzw. den gängigen Literaturempfehlungen.

## Fazit für die Praxis

- Die primäre klinische Diagnostik basiert auf dem Drawer-Test und dem Inversions‑/Eversions-Stress-Test.
- Konventionelle Röntgenbilder in 2 Ebenen werden zum Ausschluss von knöchernen Verletzungen standardmäßig durchgeführt.
- Bei Verdacht auf eine höhergradige Verletzung wird in erster Linie eine MRT-Untersuchung durchgeführt..
- Der Versorgungsstandard in Deutschland entspricht weitgehend den Empfehlungen der S1-Leitlinie der DGU bzw. den Literaturempfehlungen zur Syndesmosenverletzung.
